# The Curvilinear Relationship Between Start-Up Age and Host Growth on Sharing Accommodation Platforms

**DOI:** 10.3389/fpsyg.2022.811714

**Published:** 2022-02-09

**Authors:** Zhen Xu, Li Tang, Xintao Yu

**Affiliations:** ^1^School of Economics and Management, Tongji University, Shanghai, China; ^2^Graduate School of Technology Management, Ritsumeikan University, Osaka, Japan

**Keywords:** digital entrepreneurship, sharing accommodation, start-up age, host growth, entrepreneurial learning, product supply

## Abstract

The tourism and accommodation industry has long been a fertile field for digital entrepreneurial activities. However, sharing accommodation entrepreneurs have been ignored, whether in digital entrepreneurship or the sharing economy. This empirical study explored the relationship between start-up age and host growth based on the entrepreneurship learning theory to bridge the gap. In total, 348 hosts’ balanced panel data for 5 years were collected from the Airbnb platform. The results shown that (1) there was a curvilinear (inverted U-shaped) relationship between start-up age and host growth; (2) a critical primary growth strategy (product supply) significantly moderated the curvilinear relationship such that the inverted U-shaped relationship is less pronounced when the level of product supply is high. This study is helpful to understand digital entrepreneurs in the sharing accommodation and offers management suggestions for host growth.

## Introduction

Advances in the digital revolution have subverted the traditional business world (Kovacova et al., 2020; Kovacova and Lewis, 2021; Mas and Gómez, 2021). Against this background, the sharing economy represented by Uber, DiDi, and Airbnb has rapidly risen (Kraus et al., 2018). These sharing economy platforms manage to open up avenues for digital entrepreneurship (Ahsan, 2020). It enables consumers to become co-producers of the platform by temporarily sharing their knowledge and assets with others (usually for a fee; Sundararajan, 2014; Ahsan, 2020). Individuals who wish to start a business have found a convenient and efficient way to earn additional income. Platform users only need to click a button to become providers of various sharing platform products and services (Oskam and Boswijk, 2016). For example, ride-sharing platforms, such as Uber or DiDi, can add themselves to service providers with one click and look for sharing companions to earn additional income (Zervas et al., 2017). Thus, these sharing platforms provide adequate job creation and opportunities for those facing difficulties in bricks-and-mortar entrepreneurship (Shirazi, 2012; Novo-Corti et al., 2014; Fang et al., 2016). More and more consumers are becoming co-producers of sharing platforms, also known as digital entrepreneurs (Leick et al., 2020). According to statistics, there are estimated 84 million sharing economy service providers in China in 2020 (Center, 2021). Digital entrepreneurship under the sharing economy has gradually attracted the attention of many scholars.

Based on its low entrepreneurial threshold and labor-intensive characteristics, the tourism and hotel industry has become one of the main directions of individual digital entrepreneurship in the sharing economy (Phizacklea and Ram, 1995; Nikraftar and Hosseini, 2016). Airbnb, the world’s largest sharing accommodation platform, has become one of the main bases for individual digital entrepreneurship (Belk, 2014; Richter et al., 2015). It allows individuals to obtain, give, and share idle resources or services through the platform (Belk, 2014). Now, this platform has attracted 4 million people in more than 100,000 cities to participate in its digital entrepreneurial activities, tapping into their idle housing or becoming a real estate agent to obtain economic income (Airbnb, 2021). However, new problems emerge in the explosive growth of sharing accommodation, such as “revenue weakness” and “product homogeneity” (Voltes-Dorta and Inchausti-Sintes, 2021). As a result, most digital entrepreneurs opted out because of poor management and low efficiency (Ahmad et al., 2014; Alrawadieh and Alrawadieh, 2018; Kovacova et al., 2020). Madan et al. (2015) shows that nearly 50% of sharing economy entrepreneurs, including the Airbnb platform, only operate a shared business for 2 years or less. Therefore, one urgent question is how digital entrepreneurs in the sharing economy maintain their operations and growth?

Digital entrepreneurship is commonly considered to sell products or services through electronic networks (Guthrie, 2014), which is different from traditional entrepreneurship in many aspects (Hull et al., 2007). Over the past 2 years, scholars have carried out many studies on digital entrepreneurship. Some topics are primarily discussed, such as digital business models (Richter et al., 2017; Margiono et al., 2018), digital platforms (McIntyre and Srinivasan, 2017; Nambisan et al., 2018), and digital entrepreneurship ecosystems (Autio et al., 2018; Song, 2019). However, as a new digital entrepreneurial model, the sharing accommodation business model is not clear enough for digital platform entrepreneurs (Kraus et al., 2018). Contrary to ordinary small- and medium-sized enterprises (SMEs), the entrepreneurship of sharing accommodation is highly dependent on the entrepreneurial activities of the homeowner. Founder characteristics are considered as good indicators of their likely success in the tourism industry (Reijonen, 2008; Ahmad et al., 2014). The study by Hambrick and Mason (1984) shows that the personal characteristics of managers affect the firm’s strategic choices and performance. However, the existing research on digital entrepreneurship mainly focuses on digital entrepreneurs’ personality traits (Bandera and Passerini, 2020), while the correlation between personality traits and behavioral decision-making is largely neglected (Aldrich et al., 1999). Unlike personality-centric entrepreneurial research, entrepreneurial learning research believes that entrepreneurs can achieve good corporate performance by improving their comprehensive capabilities, capturing business opportunities, integrating resources, positioning the market, and formulating corporate development strategies (Kyndt and Baert, 2015). Therefore, this study aims to investigate entrepreneurial growth in the sharing economy based on entrepreneurial learning theory.

Growth is one of the vital essences of entrepreneurship (Stevenson and Gumpert, 1991; Sexton et al., 1997). Meanwhile, firm age significantly impacts its growth (Das, 1995; Steffens et al., 2009; Ismail and Jenatabadi, 2014). Thus, the relationship between firm age and firm growth has always been a hot topic (Variyam and Kraybill, 1992; Yasuda, 2005; Coad et al., 2016). Empirical work reveals mixed findings of this issue, with some studies finding benefits (Das, 1995; Withers et al., 2011; Kilenthong et al., 2016) and others finding the opposite effect results (Slevin and Covin, 1997; Thornhill and Amit, 2003; Harvie et al., 2010). However, the relationship between start-up age and host growth under the sharing accommodation platform is still unclear. In addition, product supply is another growth strategy pursued by firms (Mudambi and Mudambi, 2002). It helps firms achieve synergies or economies of scale (Varadarajan and Ramanujam, 1987; Tallman and Li, 1996; Delios and Beamish, 1999). However, in practice, the cost of product supply may offset the economic benefits of synergy (Hitt et al., 1994; Ilinitch and Zeithaml, 1995). Especially under the sharing accommodation platform, it is unclear how the host product supply affects the performance growth.

This study focuses on digital entrepreneurs in the sharing accommodation industry to analyze the relationship between start-up age, product supply, and host growth. We use Airbnb’s crawler panel data for 5 years to establish an empirical model to explore the host performance growth. This paper examines the following issues: (1) explore the relationship between start-up age and host growth; (2) the moderating effect of product supply. Our research results have significant contributions to theory and practice. First, we advance the literature on digital entrepreneurship in the sharing economy by using entrepreneurial learning theory and proposing a curvilinear host growth model with its predictors. Although previous studies have identified firm age as an essential factor in firm growth, there is no consensus on whether the impact is positive or negative, especially in the sharing economy. Second, we validate the moderating effect of product supply in weakening the curvilinear relationship between start-up age and host growth, which has not yet been examined. Finally, our study provides new ideas for the performance growth of sharing accommodation entrepreneurs. Entrepreneurs could adjust product supply according to their start-up age to achieve optimal performance growth.

## Theory and Model

### Start-Up Age and Host Growth

#### Entrepreneurial Learning

Entrepreneurship learning is regarded as a continuous dynamic learning process for entrepreneurs to recognize, reflect, associate, and transform experience and knowledge into functional results (Rae, 2006; Cope, 2011; Pittaway and Thorpe, 2012). It improves the effectiveness of opportunity recognition and exploitation (Shane and Venkataraman, 2000; Azoulay and Shane, 2001) and improves the ability to cope with liabilities of newness (Shepherd et al., 2000; Politis, 2005). Moreover, an entrepreneur’s experience and knowledge can be interchanged. The entrepreneur’s personal experience is transformed into knowledge, and knowledge can be used to guide the choice of new experience (Politis, 2005). Both of these are critical factors that contribute to the success of entrepreneurship (Zaheer et al., 2018). The entrepreneurship learning process helps new ventures to survive and grow (Gottschalk et al., 2017). Hughes and Morgan (2007) and Rhee et al. (2010) all proved the impact of entrepreneurial learning on firm performance through empirical research.

Based on the entrepreneurship learning theory, in the early stage of the startup, entrepreneurs face existential threats of lacking legitimacy and have poor records in creating and supporting services (Amason et al., 2006; Cai et al., 2017). To ensure survival and avoid excessive trial and error, they learn as soon as possible about the business, entrepreneurship, and market-related knowledge (Anderson and Eshima, 2013). As entrepreneurs continuously accumulate experiences, their capabilities and knowledge base will accordingly be enhanced, bolstering the rapid growth of their firms (Harvie et al., 2010; Othman and Rosli, 2011). Contrary to the young firm, older firms benefit from established practices and procedures by experience and knowledge (Freeman et al., 1983). However, these established conventions may evolve into core rigidities, hindering the willingness and ability of further entrepreneurial learning, and weakening the firm growth rate (Leonard-Barton, 1992). In general, although the research on entrepreneurial learning has been well developed in the existing literature, entrepreneurship learning research on sharing platforms is still in its infancy. Therefore, this study will discuss the entrepreneurial learning and host growth of the sharing accommodation platform based on the entrepreneurial learning theory.

#### Relationship Between Start-Up Age and Host Growth

Start-up age is defined as the number of years since initial business (Baum and Silverman, 2004; Kanze et al., 2018). New businesses always face survival pressure in the early stages of entrepreneurship and lack basic management knowledge and skills. At this time, entrepreneurial learning is an essential process for entrepreneurs (Deakins and Freel, 1998) because the decisions and strategies of a small business are directly affected by the entrepreneur (Kilenthong et al., 2016). Especially in the context of sharing accommodation, less experienced hosts lack professional market knowledge and platform services. According to the entrepreneurial learning theory, with the growth of start-up age, the host continues to learn and accumulate experience. The experience is transformed into management knowledge to understand consumer needs better, adjust marketing strategies, and make pricing decisions. Especially in the initial start-up stage, businesses urgently need to transform time and experience accumulation into management knowledge and ability through entrepreneurial learning. A large amount of entrepreneurial learning has brought about the rapid conversion from experience to knowledge. Therefore, as the start-up age moves from a young to moderate age, host growth will increase due to the transformation from learning and experience to knowledge.

However, knowledge also has adverse effects because of the limited timeliness (Lu et al., 2021). Research shows that market orientation is one of the most critical issues for start-ups (Kajalo and Lindblom, 2015; Sommer, 2018), especially in the digital economy, where market demand is short and changes rapidly. Responding quickly to changing demands has become a key factor in a host’s continuous business (Malhotra and Majchrzak, 2019). According to the view of negative learning transfer, firms with older business age tend to rely on experience from past events. They are more apt to follow templates and decision-making shortcuts, so it is more difficult for them to identify new information that is inconsistent with the accumulated knowledge (Rerup, 2005). After operating for a while, sharing accommodation hosts may get rid of the threat of existence. The motivation for continuous dynamic learning is weakened, and management knowledge is gradually challenging to keep up with the rapid changes in the market. The rigidity of management knowledge and ability can undermine firm growth (De Koning, 2003). Therefore, as the start-up age moves from moderate to older, host growth will decline due to poor management and rigid thinking.

**Figure 1:**
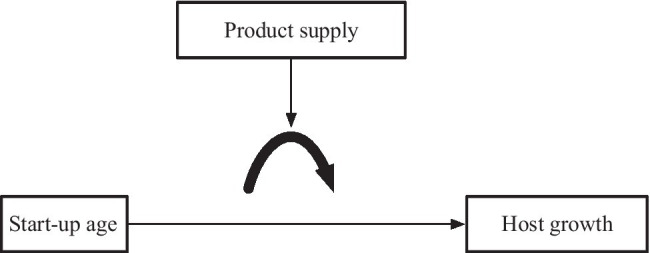
The hypothesized model.

To sum up, we believe there is a curvilinear (inverted U-shaped) relationship between start-up age and host growth. Figure 1 presents the hypothesized model of the present study. Therefore, we propose the following hypothesis:

*Hypothesis 1*. There is an inverted U-shaped relationship between a host’s start-up age and host growth such that the relationship is initially positive before an inflection point and then turns negative.

### Moderating Effect of Product Supply

Firm strategy plays a moderating role between entrepreneurial learning and firm performance (Wang, 2008). Product supply has long been regarded as one of the primary growth strategies pursued by firms (Mudambi and Mudambi, 2002). It helps firms achieve synergies or economies of scale (Varadarajan and Ramanujam, 1987; Tallman and Li, 1996; Delios and Beamish, 1999). Firms with low profitability and few growth opportunities are generally expanding through product supply (Stimpert and Duhaime, 1997). However, in practice, the cost of product supply may offset the economic benefits of synergy (Hitt et al., 1994; Ilinitch and Zeithaml, 1995). Therefore, product supply may have different effects for different firms.

When the start-up age moves from a young to moderate age, hosts spend more time learning management knowledge and capabilities without management experience and understanding of the market. In this sense, product supply brings higher management and operational challenges to firms with little experience (Oster, 2010). As a result, the ability to deal with market risks is weakened, and it is easy to face a survival crisis (Qiu, 2014). Each host runs one or more listings on the sharing accommodation platform, and each listing is diverse. For hosts, a higher level of supply means more types of listings and more customers could be served (Hutzschenreuter and Horstkotte, 2013; Qiu, 2014). As the start-up age changes from young to moderate age, managers usually have limited management experience and management capabilities, and thus are unable to cope with the large-scale listing supply. Meanwhile, product supply also leads to high costs of management resources and internal governance (Kumar et al., 2012). Therefore, when the start-up age moves from a young to moderate age, hosts with higher product supply are likely to limit the transfer of experience to knowledge and ability. This means that the curvilinear effect will be less pronounced among those with high product supply levels.

When the start-up age moves from moderate to older, the firm has reached a certain level of management experience and ability. Moreover, the emergence of the negative learning transfer effect makes it difficult for the host’s knowledge and experience to respond to the new market demands correctly. However, unlike the traditional market environment, the digital platform supports real-time matching between diverse needs and highly personalized products (Parker et al., 2016). The more rooms the host provides, the more likely it is to be retrieved by consumers. Thus, providing more rooms could expand the customer base and create a competitive advantage for a host (Hutzschenreuter and Horstkotte, 2013; Qiu, 2014). This helps hosts make up for the loss of responding to changes in demand in an untimely manner. Moreover, product supply helps reduce information asymmetry and uncertainty about product or service quality (Jean et al., 2021), thereby benefiting consumers to infer the host’s ability and reputation (Kirmani and Rao, 2000; Fernhaber and Patel, 2012). Therefore, when the start-up age moves from moderate to older, hosts with higher product supply are likely to get more customers and reduce information asymmetry to a certain extent. The curvilinear effect will be less pronounced among those with high product supply levels. Therefore, we propose the following hypothesis:

*Hypothesis 2*. The inverted U-shaped relationship between the host’s start-up age and host growth is moderated by product supply such that the effect becomes less pronounced when the level of product supply is high.

## Materials and Methods

### Data and Measures

Our data come from the world’s largest peer-to-peer accommodation sharing platform Airbnb (Nieto García et al., 2020). Airbnb has developed rapidly, with more than 5.6 million listings and 900 million arrived guests in nearly 220 countries (Airbnb, 2021). There are nearly 4 million hosts on Airbnb, and the average annual income of each host is US$9,600 (Airbnb, 2021). Considering the accuracy and objectivity of second-hand data, more and more scholars tend to use second-hand data as the data source of empirical analysis. By crawling information on the Airbnb website (i.e., listing information, customer reviews, and booking calendars), we have an opportunity to observe real business information and consumer behavior on Airbnb. In this research, we extracted all Airbnb listings information in Beijing on June 2018. We chose Beijing as the data source city as it has been widely considered to be a representative Chinese city with a leading accommodation sharing market (Wu et al., 2017; Kwok and Xie, 2018; Xie and Chen, 2019). Each Airbnb business has a unique profile page, which contains membership years, the size of the listings managed, all customer reviews, ratings and dates, and other information clues. By sorting time clues, we can track the per host’s business behavior after joining the platform. Since this research focuses on the impact of the host’s start-up age on their performance growth, we deleted hosts that did not exist between May 2013 and May 2018. The final data contain the longitudinal data of 348 hosts over a 5-year period.

Table 1 shows the definition of variables and summary statistics. The dependent variable is the growth of hosts’ review volume (*Host_growth*), which is defined as the difference between logarithm to review volume in the current period and logarithm to review volume in the previous period (Nunes et al., 2013).

**Table 1:** Descriptive statistics.

Variable	Definition	Mean	*SD*	Min	Max
*Host_growth*	Difference between logarithm to review volume in current period and logarithm to review volume in previous period	0.41	1.04	−3.00	4.41
*Age*	Number of years since a host start-up the first list	0.85	1.23	0.00	5.00
*Product_supply*	Number of rooms operated by a host	0.68	1.18	0.00	10.00
*IdentityVerified*	Dummy variable indicating whether the host has identity verifications on the Airbnb, with values of 1 = Verified, 0 = Not Verified	0.75	0.43	0.00	1.00
*ResponseTime*	The average time a host takes to response to customer reservations, with values 1 = More than a day, 2 = One day, 3 = Few hours, 4 = Less than an hour	3.49	0.85	1.00	4.00
*SuperHost*	Dummy variable indicating whether the host is recognized by Airbnb as a superhost, with values of 1 = Super Host, 0 = Regular Host	0.31	0.46	0.00	1.00
*Member*	Number of years since a host registered with Airbnb	2.46	1.63	0.00	8.00

The growth of review volume, as a measurement variable of business performance growth, usually means more popularity and booking of the host (Liang et al., 2020). There are several reasons. First, as guests can only post reviews on Airbnb after completing the booking, the hosts’ review volume reflects the lowest booking threshold. Second, Liang et al. (2017) believe that the unique design of the accommodation sharing platform makes the review volume a crucial predictor of total bookings. Third, previous research shows that most of the reviews on Airbnb are positive with ratings higher than 4.5 (the full scale is 5), so review valence is not a necessary consideration (Fradkin et al., 2015). The independent variable is the years since a host start-up his or her first list on Airbnb (*Age*). The moderating variable is the number of rooms (*Product_supply*) operated by a host. We also have control variables of host characteristics that may affect growth, including whether the host has identity verifications on the platform (*IdentityVerified*); the average time a host has taken to respond to customer reservations (*ResponseTime*); superhost status (*SuperHost*); and the years since a host joined the platform (*Member*).

### Model Specification

Because of the data structure, especially the existence of time-invariant variables, we chose the least squares dummy variable (LSDV) model with robust standard error to test. We fixed the host effect and year effect. In order to test the moderated curvilinear relationship, we followed the procedure described by Haans et al. (2016). Specifically, we focus on three basic conditions. First, the coefficients of the direct and square terms are significant, which are consistent with our hypothesis. Second, the slope of the curve at both ends of the X range is steep enough. Third, the confidence interval of the turning point is within the range of the minimum and maximum values of X. Therefore, this study analyzed two models. One is the direct curvilinear effect model, including the main and squared effect of hosts’ start-up age. The other one is the moderated curvilinear effect model, including the interaction between the product supply and the direct item as well as the interaction with the square item. This study analyzed two main equations as below:


(1) $$\begin{array}{l} Host\_growth_{it}=\alpha_{0}+\beta_{1}\log{\left(Age\right)}_{it}+\beta_{2}\log{\left(Age\right)}_{it}*\\ \log{\left(Age\right)}_{it}+\beta_{3}SuperHost_{it}+\beta_{4}IdentityVerified_{it}+\\ \beta_{5}ResponseTime_{it}+\beta_{6}Member_{it}+\mu_{i}+\lambda_{t}+\varepsilon_{it}\; \end{array}$$

(2) $$\begin{array}{l} Host\_growth_{it}=\alpha_{0}+\beta_{1}\log{\left(Age\right)}_{it}+\beta_{2}\log{\left(Age\right)}_{it}*\\ \log{\left(Age\right)}_{it}+\beta_{3}Product\_supply_{it}+\beta_{4}Product\_supply_{it}*\\ \log{\left(Age\right)}_{it}+\beta_{5}Product\_supply_{it}*\log{\left(Age\right)}_{it}*\\ \log{\left(Age\right)}_{it}+\beta_{6}SuperHost_{it}+\beta_{7}IdentityVerified_{it}+\\ \beta_{8}ResponseTime_{it}+\beta_{9}Member_{it}+\mu_{i}+\lambda_{t}+\varepsilon_{it} \end{array}$$

where $\infty_{i}$ and $\lambda_{t}$ denote the individual effects and time effects, $\alpha_{0}$ is the constant term, and $\varepsilon_{\mathrm{it}}$ represents the residual error term. We take a log transformation on the *Age* and *Product_supply* with skewed distribution. In equation (1), we estimate whether the host’s start-up age has a curvilinear impact on host growth. In equation (2), we estimate whether *product_supply* moderated the curvilinear relationship between the host’s start-up age and host growth. Since there may be multicollinearity between the interaction terms, variables in the interaction terms have been mean-centered, and models has been calculated the variance inflation factor (Day and Wensley, 1988).

## Results

Table 2 shows the correlation coefficients among all variables. First, our control variables have several significant results. Specifically, *ResponseTime* has a positive effect on *Host_growth* (β=0.133,p<0.001), *SuperHost* positively influences *Host_growth* (*β* = 0.112; *p* < 0.001), and *Member* negatively influences *Host_growth* (β=−0.114,p<0.001). Second, *Age* $\left(\beta =-0.219,\;p<0.001\right)$ and    *Product_supply* $\left(\beta =-0.048,\;p<0.05\right)$ both negatively influence *Host_growth*. Also, the variance inflation factor (VIF) of all models is tested to estimate the multicollinearity. Our result also shows that VIF values are all below 10 (with the mean at 1.48), indicating multicollinearity is not a serious problem in the study (Mason and Perreault, 1991).

**Table 2:** Correlation coefficient matrix.

	1	2	3	4	5	6	7
*Host_growth*	1.000						
*log(Age)*	−0.219^***^	1.000					
	(0.000)						
*log(Product_supply)*	−0.048^*^	0.656^***^	1.000				
	(0.073)	(0.000)					
*IdentityVerified*	0.011	0.029	−0.014	1.000			
	(0.688)	(0.225)	(0.547)				
*ResponseTime*	0.133^***^	−0.108^***^	0.020	0.019	1.000		
	(0.000)	(0.000)	(0.396)	(0.418)			
*SuperHost*	0.112^***^	0.024	−0.009	0.133^***^	0.239^***^	1.000	
	(0.000)	(0.321)	(0.712)	(0.000)	(0.000)		
*Member*	−0.114^***^	0.651^***^	0.465^***^	0.026	−0.065^***^	0.028	1.000
	(0.000)	(0.000)	(0.000)	(0.273)	(0.007)	(0.242)	

* *p* < 0.05;

*** *p <* 0.001.

Values of *p* in parentheses.

### Main Effect

Table 3 shows the results of the LSDV regression, predicting the growth of host total reviews. As the model adding independent and moderating variables, the model’s statistical coefficients (i.e., *R*^2^) increased. In model 1, control variables were introduced. The results suggest that Member has a significantly negative effect on *Host_growth* $\left(\beta =-0.121,\;p<0.001\right)$. In model 2, Age and control variables were introduced. The results suggest that Age has a significantly negative effect on *Host_growth* $\left(\beta =-1.261,\;p<0.001\right)$. In model 3, the direct effect and squared effect of Age and control variables were introduced. In addition to the Model 3 variable, Model 4 also adds *Product_supply* as the moderating variable.

**Table 3:** Main regression result.

	(1)	(2)	(3)	(4)
	Model 1	Model 2	Model 3	Model 4
*Constant*	1.873	−8.055^**^	−13.239^***^	−11.459^***^
	(3.25)	(3.18)	(3.05)	(3.00)
*IdentityVerified*	−1.011	4.922^***^	8.168^***^	7.085^***^
	(1.85)	(1.83)	(1.73)	(1.70)
*ResponseTime*	−0.368	1.953^**^	3.128^***^	2.709^***^
	(0.81)	(0.79)	(0.75)	(0.74)
*SuperHost*	1.256	−3.893^***^	−6.741^***^	−5.751^***^
	(1.50)	(1.46)	(1.47)	(1.48)
*Member*	−0.121^***^	0.263^***^	0.457^***^	0.376^***^
	(0.03)	(0.05)	(0.05)	(0.06)
*log(Age)*		−1.261^***^	0.235	0.103
		(0.17)	(0.21)	(0.56)
*log^2^(Age)*			−1.699^***^	−1.242^***^
			(0.13)	(0.38)
*log(Product_supply)*				2.619^***^
				(0.62)
log(Age)* log(Product_supply)				−4.625^***^
				(0.88)
log^2^(Age)* log(Product_supply)				1.664^***^
				(0.35)
*Host*	Yes	Yes	Yes	Yes
*Year*	Yes	Yes	Yes	Yes
*R* ^2^	0.156	0.198	0.327	0.347
adj. *R*^2^	0.128	0.072	0.099	0.123
*N*	1392	1392	1392	1392

** *p* < 0.01;

*** *p* < 0.001.

Standard errors in parentheses.

**Figure 2:**
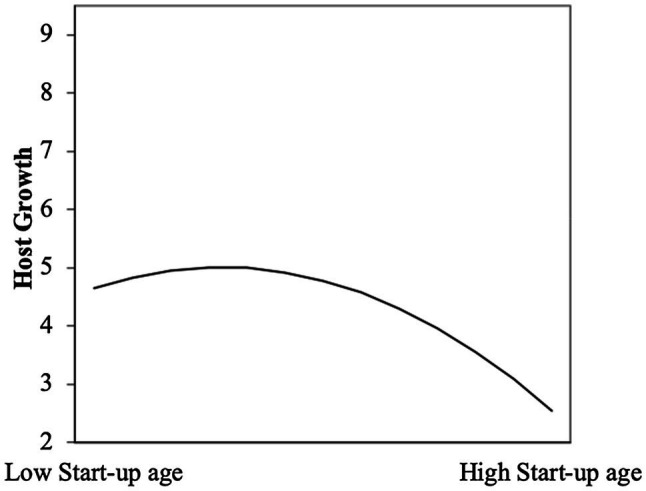
The curvilinear relationship between start-up age and host growth.

Hypothesis 1 indicates that there is a curvilinear (inverted U-shaped) relationship between start-up age and host growth. The model 3 and model 4 both show that the squared term of *Age* has a significant negative effect on *Host_growth*: Model 3 (β=−1.699,p<0.001) and model 4 (β=−1.242,p<0.001). The result shows that a curvilinear (inverted U-shaped) relationship exists between *Age* and *Host_growth*. Subsequently, a further test had shown that the slope of the curve was very steep at both ends of the X range, and the turning point of 3.5 years for Age was within the range of the minimum and maximum values (Haans et al., 2016). Therefore, as shown in Figure 2, Hypothesis 1 is supported.

### Moderating Effect of Product Supply

**Figure 3:**
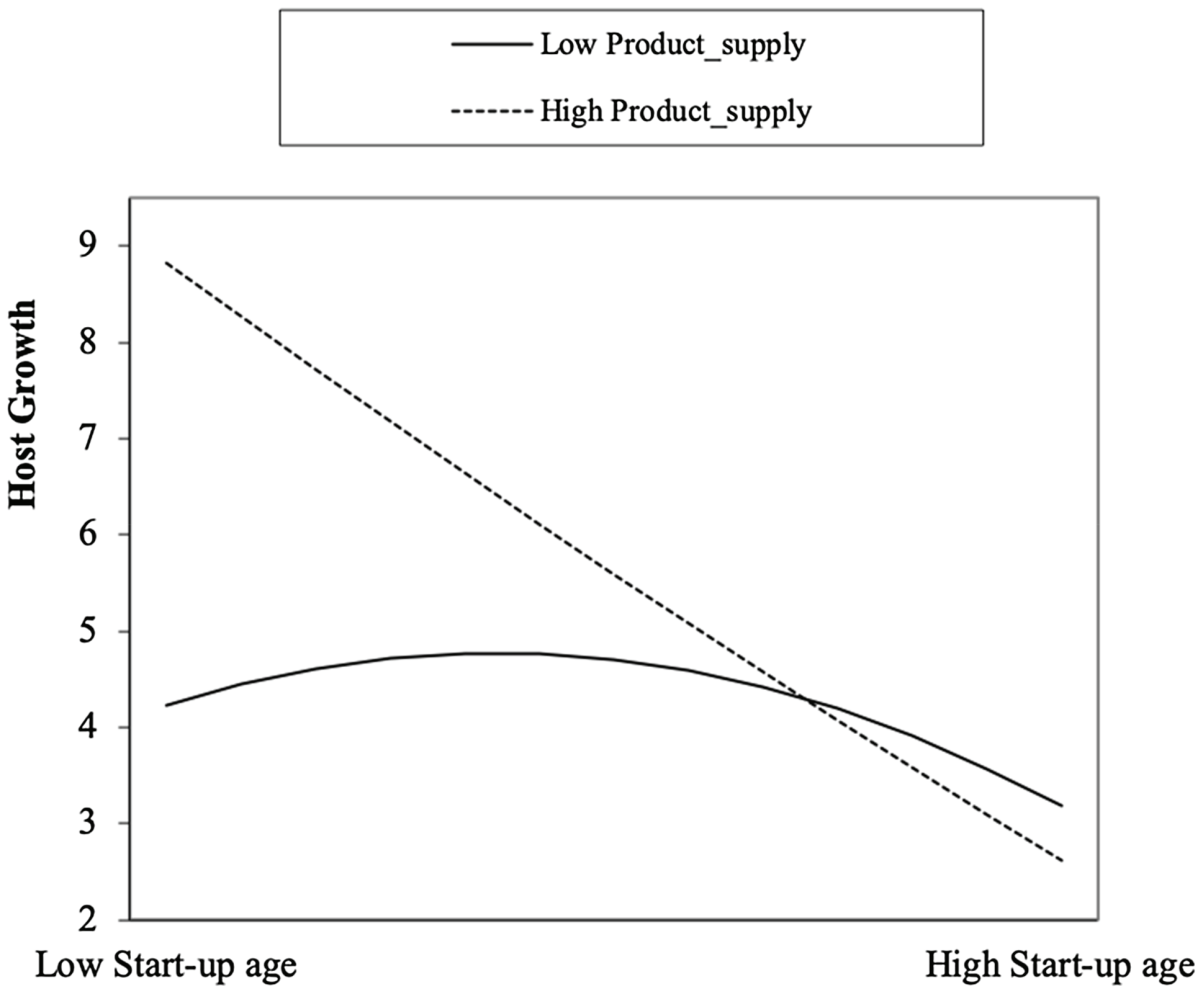
The curvilinear relationship between start-up age and host growth moderated by product supply.

Hypothesis 2 indicates that product supply can weaken the curvilinear relationship between the host’s start-up age and host growth, which means the inverted U-shaped relationship is less pronounced among host with more rent rooms compared to host with less rent rooms. Model 4 shows that the interaction term of *Age* and *Product_supply* has a significant negative effect on *Host_growth* $\left(\beta =-4.625,\;p<0.001\right)$ and the interaction term of *Age* squared and *Product_supply* has a significant positive effect on *Host_growth* $\left(\beta =1.664,\;p<0.001\right)$. These results show that the curvilinear (inverted U-shaped) relationship between Age and *Host_growth* is moderated by *Product_supply*, such that the curve is flattened when the host has more rent rooms. Figure 3 shows that there is a significant difference before and after the turning point in the simple slope coefficient of *Age* on *Host_growth* at different levels of *Product_supply*. Specifically, the main curve becomes flatter when *Product_supply* is high, which means the influence of *Age* on *Host_growth* becomes weaker. Hypothesis 2 is supported.

### Robustness Analysis

We use an alternative estimation method to be the robustness check. The robustness analysis uses pooled ordinary least squares (POLS) with robust standard errors to test without considering the individual effect and the time effect. In these POLS regression models, we also take a log transformation on *Age* and *Product_supply* with skewed distribution. As shown in Table 4, model 1 contains the control variable, model 2 adds the direct term of *Age*, model 3 adds the square term of *Age*, and model 4 adds the moderating effect of *Product_supply*. In general, the results of robustness check models are almost the same with main models, which means our results are robust.

**Table 4:** Robustness regression result.

	(1)	(2)	(3)	(4)
	Model 1	Model 2	Model 3	Model 4
*Constant*	0.144	0.120	0.038	0.111
	(0.13)	(0.13)	(0.13)	(0.13)
*IdentityVerified*	0.000	0.008	−0.008	0.006
	(0.06)	(0.06)	(0.06)	(0.06)
*ResponseTime*	0.127^***^	0.112^***^	0.106^***^	0.092^***^
	(0.03)	(0.03)	(0.03)	(0.03)
*SuperHost*	0.205^***^	0.214^***^	0.207^***^	0.220^***^
	(0.07)	(0.07)	(0.06)	(0.06)
*Member*	−0.083^***^	−0.013	−0.016	−0.042
	(0.02)	(0.03)	(0.03)	(0.03)
*log(Age)*		−0.261^***^	1.208^***^	0.138
		(0.07)	(0.18)	(0.48)
*log^2^(Age)*			−1.073^***^	−0.347
			(0.11)	(0.32)
*log(Product_supply)*				3.181^***^
				(0.55)
log(Age)* log(Product_supply)				−4.532^***^
				(0.76)
log^2^(Age)* log(Product_supply)				1.600^***^
				(0.31)
*R^2^*	0.036	0.048	0.121	0.150
*adj. R^2^*	0.034	0.045	0.117	0.145
*N*	1392	1392	1392	1392

*** *p* < 0.001.

Standard errors in parentheses.

## Discussion

The tourism and accommodation sector has been an efficient area for entrepreneurial attempts. E-entrepreneurship also becomes a vital topic of it (Oumlil and Juiz, 2018), and sharing platforms provide convenient services for entrepreneurship (Cohen and Sundararajan, 2015). However, the existing literature ignores sharing accommodation entrepreneurs. Drawing on entrepreneurship learning theory, this study investigates the impact of start-up age on host growth in the sharing accommodation sector, and the moderating effect of product supply.

First, our study indicates a curvilinear (inverted U-shaped) relationship between start-up age and host growth. This shows that start-up age promotes host growth in the early stage of start-up, and the latter stage harms host growth. This finding is consistent with the studies from the entrepreneurship learning perspective and knowledge-based view that individual experience and knowledge may be discounted (Thornhill and Amit, 2003; Desai, 2008).

Second, this study is also identified the moderating impact of product supply. As the primary growth strategies pursued by firms, product supply would weaken the influence of age on growth, both in the early and late stages of entrepreneurship. In the early stage of entrepreneurship, product supply adds operating and cost pressures to start-ups (Muñoz-Bullón and Sanchez-Bueno, 2011). However, in the late stage of entrepreneurship, product supply has become a risk reduction strategy, which is also proven by the research of Lynn and Reinsch (1990) and Rosa and Scott (1999).

### Theoretical Implications

This research may provide several novel contributions.

First, it contribute to the digital entrepreneur’s literature by highlighting the entrepreneurship learning perspective, in contrast to the research on personality traits of digital entrepreneurs (Bandera and Passerini, 2020). In doing so, this study echoes the call of entrepreneurial research in the context of the digital economy. Digital entrepreneurs must be familiar with the frequently updated rules of the platform and understand the rapidly changing and diversified needs of consumers (Shemyatikhina et al., 2020). The findings of this study show that continuous dynamic entrepreneurial learning is essential for digital entrepreneurs. Therefore, this study enriches the existing literature on digital entrepreneurs.

Second, the present study contributes to the research on entrepreneurial growth. Entrepreneurship research is concerned with establishing enterprises and how to make firms survive. Following the pioneering research of Birch (1979), the mainstream view is that young firms tend to grow faster than old firms to survive (Jovanovic, 1982; Geroski and Gugler, 2004). However, subsequent research did not reach an agreement on this issue (Harvie et al., 2010; Withers et al., 2011; Kilenthong et al., 2016). Especially for the related theories of the small business growth process are not yet mature enough (Deakins and Freel, 1998). Specific to the hotel industry, digital entrepreneurship has non-standard characteristics in sharing accommodation. It is highly dependent on the entrepreneurs themselves, and there is more significant heterogeneity with traditional hotels (Alrawadieh and Alrawadieh, 2018). However, it is difficult for general entrepreneurial growth theory results to provide consistent theoretical guidance for sharing accommodation entrepreneurship practice (Alrawadieh and Alrawadieh, 2018). Our research further extends entrepreneurial research to the sharing accommodation industry in the context of digital entrepreneurship. It clarifies the U-shaped relationship between the accumulation of knowledge over time and host growth. Thus, this study expands and enriches the mechanism of the effect of entrepreneurial learning on the growth of new ventures.

Third, our research has some enlightenment for developing upper echelons theory and the strategic management of SMEs in the tourism industry. Upper echelons theory proposes that entrepreneurs make strategic choices based on their characteristics, ultimately affecting firm performance (Neely et al., 2020). We not only examined the impact of start-up age on host growth but also investigated the moderating effect of product supply. It expands the application environment of upper echelons theory. Moreover, our study makes up for the lack of individual characteristics to explain the phenomenon of sharing economy entrepreneurship and provides a new perspective to discuss product supply issues. On the one hand, when the start-up age moves from a young to moderate age, hosts with higher product supply may limit the transfer of experience to knowledge and ability. On the other hand, when the start-up age moves from moderate to older, hosts with higher product supply are likely to get more customers and reduce information asymmetry to a certain extent. In general, this curvilinear effect would be less pronounced among those with high product supply levels. Thus, this research reveals how entrepreneurs’ personal characteristics and product supply strategies affect performance growth, providing new perspectives and ideas for entrepreneurship research on SMEs in the tourism industry.

### Practical Implications

This research also has provided some practical implications.

First, for the sharing accommodation platform designer, this study reveals some insights into supporting hosts to provide continued services. Our results indicate that entrepreneurial learning is vital to the hosts. The lack of comprehensive management capabilities of entrepreneurs is one of the crucial reasons for entrepreneurial failure (Amjad, 2020). In the early stage of hosts’ entrepreneurship, the platform designer should provide them with training courses or practical online management tools to help entrepreneurs establish standardized operations in their daily operations, thereby accumulating operation management and service marketing-related knowledge, experience, and skills. In the late stage of hosts’ entrepreneurship, senior hosts should be encouraged to manage multiple houses. This will encourage them to understand new market knowledge, break their long-standing “tacit understanding” of the industry’s success secrets, and resist the risk of incorrect empirical judgments.

Second, for hosts, the obtained results indicate that experience and knowledge are the crucial factors for hosts to improve their online income growth. More in-depth entrepreneurial learning can undoubtedly lead to an increased online income. Our research finding is significant for those junior hosts who lack entrepreneurial or rental experience. Since junior hosts do not have enough experience and skills to ensure the management of listing, so it is not recommended for hosts to seek high-level product supply when the start-up age moves from low to moderate levels. However, to get more customers and reduce information asymmetry, senior hosts could seek high-level product supply when the start-up age moves from moderate to high levels. This study is also instructive for SMEs. The continuous learning process can continuously provide new knowledge and information for start-ups so that start-ups survive and grow in a turbulent environment. Thus, new ventures must focus on learning in entrepreneurial practice.

Third, this research can also provide implicature for reducing the failure rate of digital startups and stimulating the entrepreneurial activities of local SMEs. The hotel and tourism industry, especially for small and medium hotel and tourism firms, is a crucial engine of economic growth in many countries, with its growth rate exceeding that of many other industries (Carlisle et al., 2013; Tang and Tan, 2013; Webster and Ivanov, 2014; Hallak et al., 2015). However, the failure rate of new ventures is generally high (Amjad et al., 2020a,b,c; Dutta and Sobel, 2021). To maintain the development and expansion of the industry, the government should organize digital entrepreneurs to conduct excellent entrepreneurial experience learning, help local tourism entrepreneurs to build confidence in their capabilities, provide supporting services and training programs, and ultimately stabilize and promote local employment and economic development.

### Limitations and Future Research Directions

This study bears several limitations. First, only a single city is selected for empirical analysis. Thus, the sample size of this study is relatively small. Since the fitting coefficient is sensitive to the sample size, a large sample would be beneficial to future research works. Future research can expand the sample to a wider area to verify the universality of the conclusions of this research. Second, this study considers the issue of the relationship between age and host growth only from an entrepreneurship learning perspective. However, age is also regarded as a quality signal under the signal theory perspective. Such perspective could be included in future studies to gain additional insights. Finally, some important variables, such as part-time or professional host, were not included in the control variables. Professional hosts could have sufficient time to learn and interact with consumers, affecting the accumulation of host-related experience and knowledge. However, it is not easy to obtain these data from the Airbnb website because it is not marked explicitly in Airbnb’s public data. Consequently, it may be one potential limitation of this study.

## Conclusion

The sharing economy provides numerous opportunities and a suitable platform for digital entrepreneurship (Cohen and Sundararajan, 2015). However, previous studies neglect the digital entrepreneur research in the field of sharing accommodation (Kraus et al., 2018). Based on the entrepreneurship learning theory, this study investigates the impact of start-up age on host growth in the sharing recommendation industry. We verify our hypotheses by using data from 348 hosts on Airbnb. We offer clarity that the effect of start-up age on host growth is curvilinear. Moreover, we extend theory by introducing a key primary growth strategy (product supply) and linking product supply with start-up age to better explain host growth. This research not only enriches the empirical research understanding of digital entrepreneurship in the sharing economy, but also provides practical enlightenment for the host to decide whether to implement product supply based on their entrepreneurial age.

## Data Availability Statement

Publicly available datasets were analyzed in this study. This data can be found at: http://insideairbnb.com/get-the-data.html.

## Author Contributions

ZX and LT contributed to the current research ideas and design of this study. LT wrote the first draft of the manuscript. ZX performed the statistical analysis and contributed to improve the manuscript. XY edited the revised manuscript and contributed to avoid language errors. All the authors contributed to the article and approved the submitted version.

## Conflict of Interest

The authors declare that the research was conducted in the absence of any commercial or financial relationships that could be construed as a potential conflict of interest.

## Publisher’s Note

All claims expressed in this article are solely those of the authors and do not necessarily represent those of their affiliated organizations, or those of the publisher, the editors and the reviewers. Any product that may be evaluated in this article, or claim that may be made by its manufacturer, is not guaranteed or endorsed by the publisher.
